# Phosphorylation of NLGN4X Regulates Spinogenesis and Synaptic Function

**DOI:** 10.1523/ENEURO.0278-23.2025

**Published:** 2025-03-12

**Authors:** Alexander W. Lehr, Thien A. Nguyen, Wenyan Han, Eunhye Hong, John D. Badger, Wei Lu, Katherine W. Roche

**Affiliations:** ^1^Receptor Biology Section, National Institute of Neurological Disorders and Stroke, National Institutes of Health, Bethesda, Maryland 20892; ^2^Department of Neuroscience, Brown University, Providence, Rhode Island 02906; ^3^Department of Pharmacology and Physiology, Georgetown University, Washington DC 20057; ^4^Synapse and Neural Circuit Section, National Institute of Neurological Disorders and Stroke, National Institutes of Health, Bethesda, Maryland 20892

**Keywords:** autism spectrum disorder, neuroligin, phosphorylation, sex-linked, spine morphology, spinogenesis

## Abstract

Neuroligins (NLGNs) are a family of postsynaptic adhesion molecules that bind to their presynaptic partners, neurexins, facilitating the formation and maintenance of synapses. In humans, there are five genes encoding NLGNs (*NLGN1-3*, *NLGN4X*, and *NLGN4Y*), with *NLGN1-3* having highly conserved counterparts in rodents, allowing these genes to be studied with high confidence of translational validity in mouse models. Human NLGN4X and 4Y were often assumed to serve similar functions because they share a 97% sequence homology, whereas mouse NLGN4-like is quite divergent. Many NLGN-mediated synaptic effects are modulated through post-translation modifications, which exert temporal and spatial control. In this report, we characterize a conserved phosphorylation site, serine 712, on NLGN4X and 4Y. Despite serine 712 being located in a highly conserved region between NLGN4X and 4Y, we observed kinase specificity. PKA exclusively phosphorylates NLGN4X S712, whereas Cdk5 phosphorylates S712 on both NLGN4X and 4Y. NLGN4X S712 phosphorylation regulated spine density, with phosphorylation reducing mature mushroom spines and unphosphorylated S712 increasing spines and enhancing miniature excitatory postsynaptic current frequency.

## Significance Statement

Phosphorylation is a key regulator of synaptic function, allowing changes in protein–protein interactions and switching of protein function through the addition of a highly charged phosphate group. Neuroligins (NLGNs) are important neuronal adhesion molecules that mediate synaptic development, maintenance, and plasticity. We have now discovered analogous phosphorylation sites on the X–Y paired genes of *NLGN*, *NLGN4X* and *NLGN4Y*, which are differentially phosphorylated by PKA and Cdk5. In addition, our results reveal NLGN4X phosphorylation at this site regulates spinogenesis and spine maturation. Going forward, more research is needed to determine the role sex-linked NLGNs perform at the synapse in both physiological conditions and in neurodevelopmental disorders.

## Introduction

Neuroligins (NLGNs) are postsynaptic adhesion proteins discovered through their interaction with presynaptic neurexins facilitating the close contact of the two synaptic membranes (Scheiffele et al., 2000; Sudhof, 2008; Bemben et al., 2015a; Jeong et al., 2017). In humans, NLGNs are encoded by five genes*, NLGN1-3*, *4X*, and *4Y*, each with distinct expression patterns and functions. For example, NLGN1 localizes to excitatory synapses, whereas NLGN2 is found at inhibitory synapses, and NLGN3 is expressed at both (Chih et al., 2004; Graf et al., 2004; Chubykin et al., 2007; Poulopoloulos et al., 2009; Bemben et al., 2015b; Chanda et al., 2017). NLGNs 1 through 3 are highly conserved between humans and common model organisms like rat and mouse, allowing them to be studied with a high level of translational confidence, whereas NLGN4 is highly divergent between human and mice (Bolliger et al., 2008). *NLGN4X* and *4Y* reside in the differential region of the sex chromosomes, having arisen as an X–Y pair recently in mammalian evolution accompanying the degradation of the pseudoautosomal region of the sex chromosomes (Maxeiner et al., 2020; Nguyen et al., 2020b). NLGN4X is found at glutamatergic synapses in humans, in contrast to mouse NLGN4-like, which is found at glycinergic synapses (Hoon et al., 2011; Zhang et al., 2018). Additionally, mouse NLGN4-like is located within the pseudoautosomal region of the sex chromosome and only shares ∼60% homology to human *NLGN4X/Y*.

At the synapse, protein kinases serve as an important intermediary between synaptic activity and NLGN function. For instance, surface expression of NLGN1 is modulated by Ca^2+^ calmodulin-dependent kinase II (CaMKII) phosphorylation at T739; postsynaptic density (PSD)-95 binding and endocytosis are modulated by PKA phosphorylation at S839; gephyrin binding is controlled by phosphorylation at Y782 (Bemben et al., 2014; Giannone et al., 2013; Letellier et al., 2018; Jeong et al., 2019). NLGN4X is phosphorylated by protein kinase C (PKC) at T707, and this phosphorylation event results in increased excitatory synaptic transmission (Bemben et al., 2015a). Of note, a rare variant near this phosphorylation site, R704C, was discovered in a male ASD patient, with an unaffected sister carrying the same R704C variant (Yan et al., 2005). Additional characterization revealed the ASD-associated mutant NLGN4X R704C cannot be phosphorylated by PKC at T707 and is thus unable to enhance synaptic transmission via this PKC-dependent mechanism (Bemben et al., 2015a). Taken together, phosphorylation is a dynamic mechanism to regulate and modulate NLGN function.

In this study, we identify a conserved phosphorylation site on NLGN4X and NLGN4Y, serine 712, and characterize it as a regulator of dendritic spine maturation. We show differential phosphorylation of NLGN4X and NLGN4Y at S712, depending on the kinase. Importantly, kinase specificity can be swapped through a single amino acid exchange between NLGN4X and NLGN4Y. Our characterization indicates that NLGN4X S712 is an important regulator of synaptic spine development and expands to the known role of kinases in isoform-specific NLGN regulation.

## Materials and Methods

### Plasmids and antibodies

Human pCAG-HA-NLGN4X (WT, S712A, S712D, or R704C)-IRES-mCherry and pCAG-NLmiRs-GFP plasmids were used for biochemical, electrophysiological, and imaging experiments. pGEX-GST-NLGN ICD constructs were made as previously described (Bemben et al., 2014). To generate the rabbit NLGN4X/4Y pS712-Ab (against residues 709–718 in NLGN4X and 4Y), we immunized the animals with synthetic phosphopeptide Ac-RRP(pS)PQRNTT-amide (New England Peptide). All immunoblotting with NLGN4X/4Y pS712-Ab begins with blocking in 5% PhosphoBLOCKER (Cell Biolabs, AKR-103) in TBS-T at room temperature, followed by 1% PhosphoBLOCKER in the primary and secondary antibody incubations. Antibodies used in the study were anti-NLGN4X (Sigma-Aldrich, sab1407790), anti-NLGN4X (Abcam, ab181251), anti-GST (Bethyl Laboratories, A190–122A), anti-HA rat (Roche, 11867423001), anti-HA rabbit (Abcam, ab9110), anti-PSD-95 (NeuroMab, 75-028), anti-actin (Applied Biological Materials, G043).

### GST-fusion protein production and in vitro phosphorylation

Reagents were prepared and assays were performed and analyzed as previously described (Bemben et al., 2014).

### Neuronal differentiation from iPSCs

Neural stem cells (NSCs) were differentiated from induced pluripotent stem cells (iPSCs) using the simplified doxycycline-inducible NGN2 stable hiPS cell line as described in Wang et al. (2017). hiPS cells predifferentiated into NSCs via a 3 d treatment with doxycycline were then replated. Drug treatment and cell collection were performed 3 weeks after plating, at days in vitro (DIV)21. Cells were lysed in 1% Triton X-100 buffer, and cell lysates were incubated overnight with NLGN4X/4Y pS712-Ab and bound the subsequent day onto Protein-A beads for 1 h. The immunoprecipitated samples were washed in TBS buffer containing 150 mM NaCl, 50 mM Tris⋅HCl, 1 mM EDTA, as well as protease (Roche, 11836145001), and phosphatase inhibitors (Sigma-Aldrich, P5726, P0044). The sample proteins were resuspended in SDS/PAGE sample buffer, resolved by SDS/PAGE, and immunoblotted.

### Neuronal cultures

Primary neuronal cultures were prepared from E18 Sprague Dawley rats and used for immunocytochemistry and phosphatase experiments. The use and care of animals used in this study followed the guidelines of the National Institutes of Health Animal Research Advisory Committee. Hippocampal neurons were plated onto glass coverslips precoated with poly-d-lysine (Sigma-Aldrich, P7280). Hippocampal neurons were cotransfected with Lipofectamine 2000 (Invitrogen, 11668-019) at DIV13 with NLmiRs and HA-NLGN4X constructs and then fixed and labeled on DIV17. Cortical neurons were plated onto poly-d-lysine–coated 10 cm tissue culture dishes at a concentration of nine million cells per dish. Primary cortical neurons were transfected with Lipofectamine 2000 at DIV5 with HA-NLGN4X WT construct, and cells were collected on DIV7, lysed in 1% Triton X-100, sonicated, and used for phosphatase treatment experiments.

### Immunocytochemistry

Surface trafficking was assessed via staining surface versus intracellular HA-tagged NLGN and measuring intensities of both channels from two to three regions per secondary dendrite. To label surface proteins, live cells were incubated with primary rat HA antibody (Roche, 1186742300) at room temperature for 10 min in neurobasal media. Cells were then washed in PBS, fixed for 10 min in a 4% paraformaldehyde and 4% sucrose PBS solution, and washed again. Cells were blocked in 10% normal goat serum (NGS; Vector, S1000) and surface stained with Alexa Fluor 555-conjugated anti-rat secondary antibody (Invitrogen, A-21434) in 3% NGS. Cells were permeabilized with 0.25% Triton X-100 for 10 min, washed with PBS, and then intracellularly probed using rabbit HA antibody (Abcam, ab9110) primary and GFP (Invitrogen, A10262) to augment the signal from the GFP-fill from the NLmiRs construct and stained with Alexa Fluor 647-conjugated anti-rabbit (Invitrogen, A-21245) and Alexa Fluor 488-conjugated anti-chicken (Invitrogen, A-11039) secondary antibodies. Blinded analysis of the NLGN4X (*n* = 25), NLGN4X S712A (*n* = 24), and NLGN4X S712D (*n* = 27) conditions were averaged, and a one-way ANOVA was performed, comparing between NLGN4X WT and phosphomutant conditions.

### Automated spine classification

Neurons were stained for HA, GFP, and the excitatory postsynaptic marker PSD-95 (which was not used for analysis in this study and is thus not depicted). Imaging for automated spine morphology analysis was optimized according to a previous report (Dickstein et al., 2016). Automatic spine detection and quantification were performed blinded on one to three regions of secondary dendrites per neuron using the GFP cytoplasmic fill channel with the Neurolucida 360 (MBF Bioscience) software, on NLGN4X (*n* = 44 regions), NLGN4X S712A (*n* = 50 regions), NLGN4X S712D (*n* = 57 regions), and NLGN4X R704C (*n* = 44 regions) conditions. Detection settings in Neurolucida 360 for three-dimensional reconstruction of spines were set as follows: outer range, 5 μm; minimum height, 0.3 μm; detector sensitivity, 130%; and minimum count, 10 voxels. Reconstructed spines were then manually inspected to ensure accuracy, with errant spines removed or manually reconstructed. Spines were then characterized using default setting in Neurolucida 360 (head-to-neck ratio, 1:1; length-to-head ratio, 2:5; mushroom head size, 0.35 μm; filopodium length, 3 μm).

### Phosphatase treatment

Lysate from primary cortical neurons transfected with HA-NLGN4X was treated with lambda protein phosphatase. Protein concentration was measured, and 1 μg of protein was treated with 2 μl of lambda protein phosphatase (New England Biolabs, P0753S), incubated at 30°C for 30 min, resolved by SDS/PAGE, and immunoblotted.

### Electrophysiological recording

The miniature excitatory postsynaptic current (mEPSC) recordings were performed in dissociated rat hippocampal primary cultures (15–17DIV). Recordings were done in artificial cerebrospinal fluid (ACSF) containing (in mM) 119 NaCl, 2.5 KCl, 26 NaHCO_3_, 1 Na_2_PO_4_, 11 glucose, 2.5 CaCl_2_, and 1.3 MgCl_2_; 0.1 mM picrotoxin 0.1 mM and 0.5 μM TTX were added to the ACSF before recording. The intracellular solution for mEPSC recording contained the following (in mM): 135 CsMeSO_4_, 8 NaCl, 10 HEPES, 0.3 Na3GTP, 4 MgATP, 0.3 EGTA, and 5 QX-314. The osmolality of the solutions was adjusted from 285 to 290 mOsm, and pH was buffered from 7.25 to 7.35. The mEPSCs were recorded at −70 mV, and the analysis of the mEPSCs was done semiautomatically, using the in-house software Igor Pro (WaveMetrics) developed in Roger Nicoll's laboratory at the University of California, San Francisco. All events were visually inspected to ensure they were mEPSCs during analysis, and those noncurrent traces were discarded. Series resistance was monitored and not compensated, and cells in which series resistance varied by 25% during a recording session were discarded. Synaptic responses were collected with a MultiClamp 700B amplifier (Axon Instruments), filtered at 2 kHz, and digitized at 10 kHz. All pharmacological reagents were purchased from Abcam, and other chemicals were purchased from Sigma-Aldrich.

### Statistical analysis

Data analysis was conducted using ImageJ, Neurolucida360, and GraphPad Prism software. Experiments were performed blinded at least three independent times. Significant differences in phosphorylation levels were determined via conventionally utilized *t* test comparisons. For spine morphology analysis, each neuron had 1–3 secondary dendrites classified, with all individual dendrites being averaged and one-way ANOVA performed. Significance for cumulative probability distributions in electrophysiology experiments was assessed using the Kolmogorov–Smirnov normality test with Dallal–Wilkinson–Lillie for the *p* value. Statistical significance is defined as *p < 0.05, **p < 0.01, ***p < 0.001, and ****p < 0.0001.

## Results

### PKA phosphorylates NLGN4X at S712

**Figure 1.**
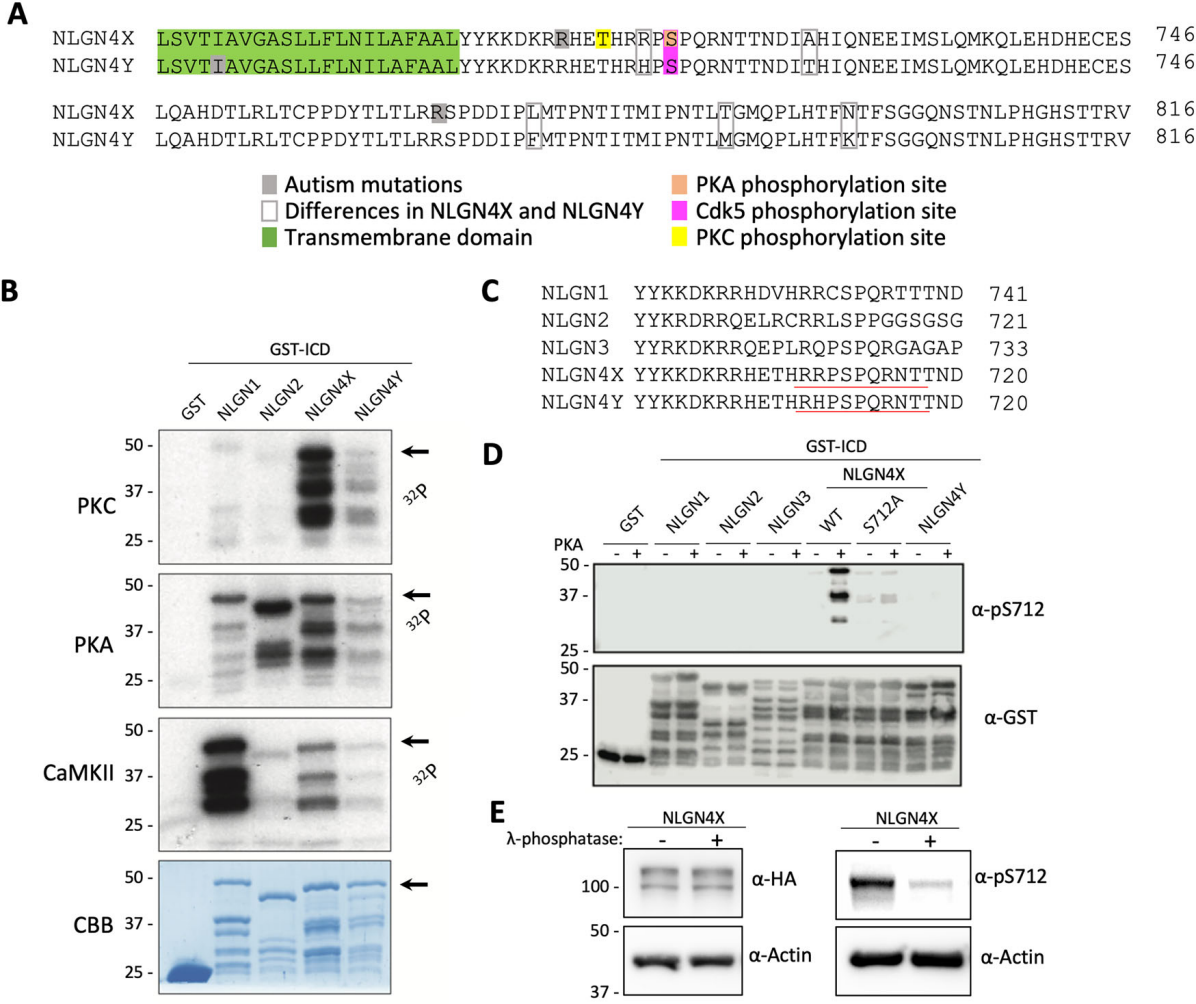
NLGN4X is phosphorylated by PKA at S712. ***A***, Alignment of the transmembrane membrane domain and ICD of human NLGN4X and NLGN4Y. The PKC phosphorylation site, T707, is boxed in yellow; ASD mutations are boxed in gray; the transmembrane domain is boxed in blue; the PKA site on NLGN4X S712 is boxed in green; and the Cdk5 phosphorylation site on NLGN4X and NLGN4Y S712 is boxed in pink. ***B***, GST-fusion proteins were incubated with [γ−^32^P] ATP and purified PKA, PKC, and CaMKII. Phosphorylation of NLGNs was analyzed by autoradiography. CBB protein staining was used as the loading control. The arrow indicates the size of the undegraded form of GST-fused NLGN4X C-tail. ***C***, Sequence alignment of NLGN1–3, 4X, and 4Y. The NLGN4X phosphorylated S712 antibody epitope is underlined in red. ***D***, GST-fusion proteins of NLGN1–3, 4X (WT, S712A), or 4Y were incubated with purified PKA. PKA phosphorylation was analyzed by immunoblot probing with pS712 antibody. ***E***, Lysate from transfected primary cortical neurons was treated with lambda protein phosphatase and evaluated by immunoblotting with HA-Ab or pS712-Ab. α-Actin was used as a loading control.

Our group has previously demonstrated that PKC phosphorylates NLGN4X at T707 (Bemben et al., 2015a; Fig. 1*A*). To characterize other phosphorylation sites on NLGN4X and 4Y, we used an in vitro kinase assay, with GST-fusion intracellular domains (ICDs) of NLGNs to screen for phosphorylation by several neuronal kinases (PKC, PKA, and CaMKIIα). We observed that PKC (as previously studied in Bemben et al., 2015a), PKA, and CaMKII robustly phosphorylated NLGN4X, but not 4Y (Fig. 1*B*). Interestingly, there is a conserved residue among all NLGN genes, which has been identified as a substrate for PKA at the analogous residue S714 in NLGN2 (Antonelli et al., 2014; Halff et al., 2022) and Cdk5 at NLGN3 S725 (Jeong et al., 2023) but is uncharacterized in NLGN4X. To study the phosphorylation of NLGN4X at the analogous S712, we developed a phosphorylation state-specific antibody using residues 709–718 as the epitope (Fig. 1*C*). Using an in vitro kinase assay, we validated our antibody's specificity against all NLGN ICDs as well as a phospho-dead, serine to alanine mutant of NLGN4X (S712A). We observe that our antibody robustly detects NLGN4X phosphorylated by PKA while minimally detecting the phosphonull S712A mutant, demonstrating our antibody as highly phosphospecific when compared with other phosphorylation state-specific antibodies (Fig. 1*D*). To confirm that our antibody recognizes phosphorylated NLGN4X, we overexpressed NLGN4X in cortical neurons, applied phosphatase treatment to the lysate, and observed a dramatic reduction of pS712 signal when compared with basal phosphorylation levels (Fig. 1*E*).

**Figure 2.**
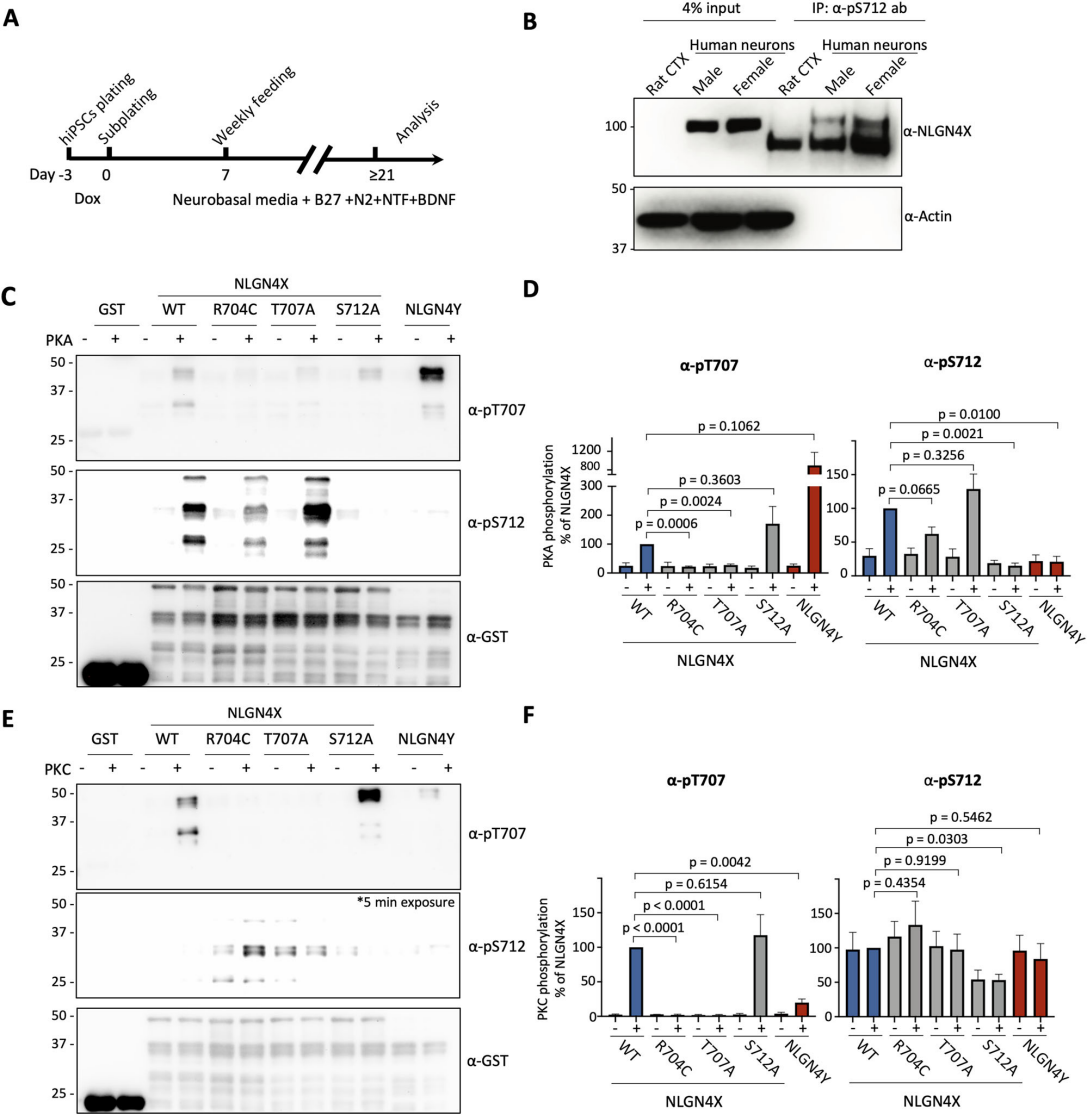
NLGN4X is expressed and phosphorylated at S712 in human neurons. PKA and PKC are specific to phosphorylating NLGN4X S712 and NLGN4X T707, respectively. NLGN4X R704C ASD mutation disrupts NLGN4X S712 phosphorylation. ***A***, Schematic for generating induced neurons from hiPS cells. ***B***, Induced neurons from hiPS cells were immunoprecipitated with NLGN4X/4Y pS712 antibody. Phosphorylation of NLGN4X was evaluated by immunoblot, probing with NLGN4X antibody. ***C***, ***E***, GST-fusion proteins of NLGN4X (WT, R704C, T707A, S712A) and NLGN4Y (WT) were incubated with PKC or PKA, and phosphorylation was evaluated by immunoblotting with pT707-Ab or pS712-Ab. Total protein was evaluated with GST-Ab. ***D***, ***F***, Phosphorylation levels (mean ± SEM) were normalized to NLGN4X. Statistical significance was tested using a *t* test (*n* = 3).

To ensure that human endogenous NLGN4X is phosphorylated at S712, we utilized differentiated neurons from human iPSCs and observed phosphorylation using our phosphospecific antibody (Fig. 2*A*). We immunoprecipitated phosphorylated NLGN4X using our phosphospecific antibody and detected its enrichment via blotting against NLGN4X (Fig. 2*B*).

To determine if PKA and PKC phosphorylation were specific to their respective phosphorylation sites, we conducted additional in vitro *kinase* assays. Through this, we demonstrate that PKA can phosphorylate T707A, whereas PKC does not. Also, respectively, PKC can phosphorylate S712A, whereas PKA does not, demonstrating these kinases are specific to their respective phosphorylation sites (Fig. 2*C*–*F*). We’ve previously shown that PKC phosphorylation of NLGN4X T707 is disrupted by the ASD-associated mutation R704C (Bemben et al., 2015a). In this current study, we demonstrate that the R704C mutation does not significantly disrupt the nearby PKA phosphorylation of S712 (Fig. 2*C*,*D*). It is important to note that while the R704C has been studied as an ASD-associated mutation and was recently deposited into ClinVar, this mutation does appear in many unaffected individuals in gnomAD (most of which are female).

### Kinase specificity of NLGN4X and NLGN4Y is dictated by a single residue substitution

**Figure 3.**
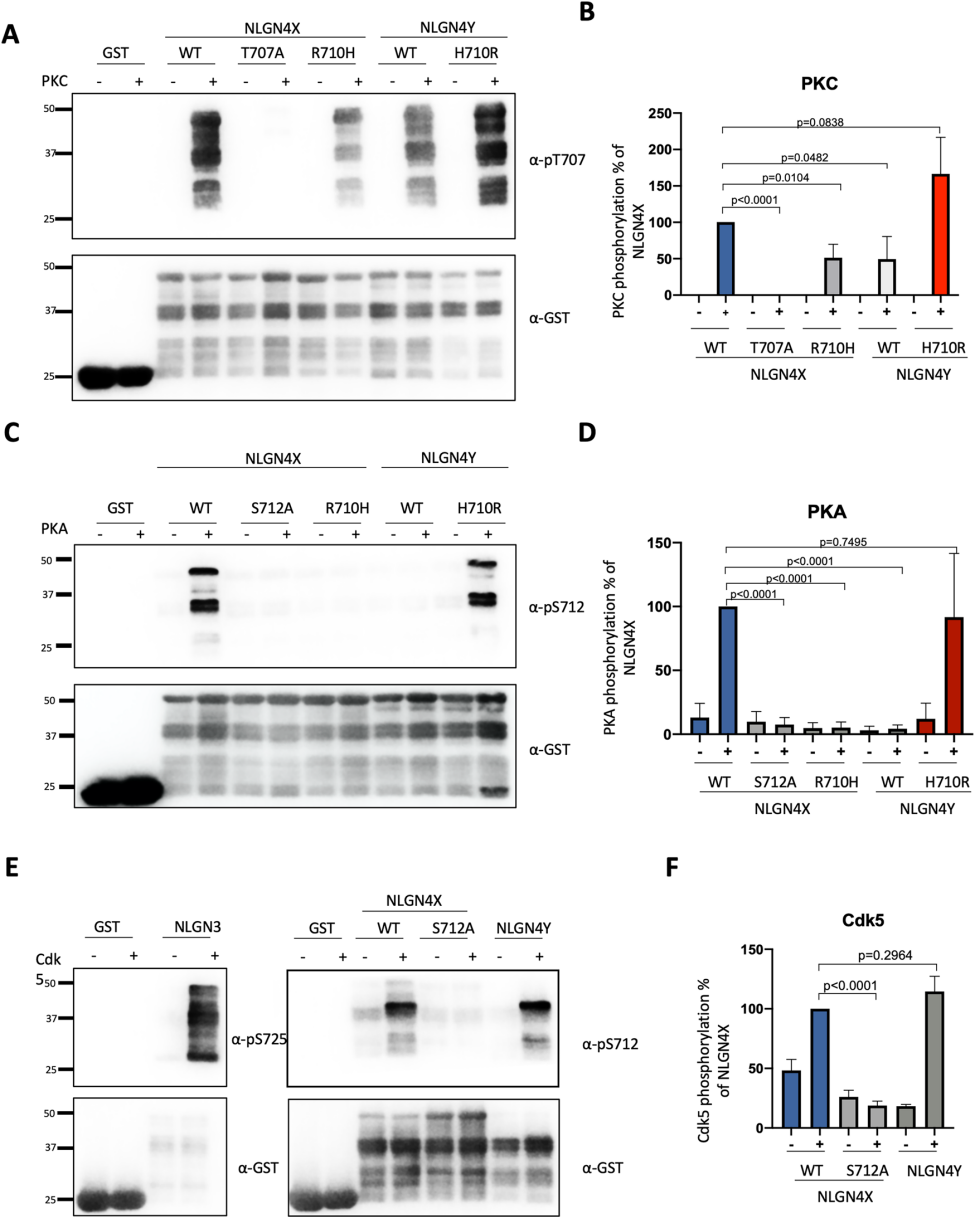
Serine 712 phosphorylation is conserved in NLGN4Y but shows kinase specificity. ***A***, ***C***, ***E***, GST-fusion proteins of NLGN4X (WT, T707A, R710H, S712A) and NLGN4Y (WT, H710R) were incubated with PKC, PKA, or Cdk5, and phosphorylation was evaluated by immunoblotting with pT707-Ab or pS712-Ab. Total protein was evaluated with GST-Ab. ***B***, ***D***, ***F***, Phosphorylation levels (mean ± SEM) were normalized to NLGN4X. Statistical significance was tested using a *t* test (*n* = 4).

NLGN4X and 4Y have very high sequence conservation, making the differential phosphorylation surprising. We wanted to understand the molecular basis for NLGN4Y acting as a poor substrate of PKC and PKA on the conserved T707 and S712 sites as compared with NLGN4X. Examination of the NLGN4X and NLGN4Y sequences revealed one amino acid difference between the two isoforms at position 710, an arginine (R710) on NLGN4X, and a histidine (H710) on NLGN4Y. This position piqued our interest due to its intriguing location between the PKC phosphorylation site (T707) and PKA phosphorylation site (S712). We hypothesized this differential residue dictates the phosphospecificity of PKA and PKC acting exclusively on NLGN4X. Thus, we made GST-ICD NLGN4X and NLGN4Y constructs, swapping their respective 710 residues (NLGN4X R710H and NLGN4Y H710R) to determine if that would also shift the phosphospecificity of our kinases, giving PKA and PKC the ability to phosphorylate NLGN4Y and not NLGN4X. Using an in vitro kinase assay, we showed that NLGN4X R710H (containing the respective NLGN4Y histidine) was not phosphorylated by PKA and PKC. In contrast, NLGN4Y H710R can be robustly phosphorylated by both PKA and PKC (Fig. 3*A*–*D*). Together, we show that an amino acid difference between NLGN4X and NLGN4Y can account for the differential phosphorylation pattern of the two highly conserved proteins.

### Cdk5 phosphorylation of NLGN4X and NLGN4Y at S712

In investigating the selective phosphorylation of NLGN4X by other kinases using in vitro kinase assays on GST-fusion ICD, we discovered that both NLGN4Y and NLGN4X were robustly phosphorylated by Cdk5 (Fig. 3*E*,*F*). We used phosphorylation of NLGN3 as a positive control for Cdk5 phosphorylation at its analogous S725 site. We thus confirmed that NLGN4X and NLGN4Y are phosphorylated at S712, unlike PKA, which only phosphorylates NLGN4X and not NLGN4Y. Thus, there is a kinase and isoform specificity to the S712 phosphorylation site.

### Phosphorylation of NLGN4X at S712 decreases dendritic spine maturation

**Figure 4.**
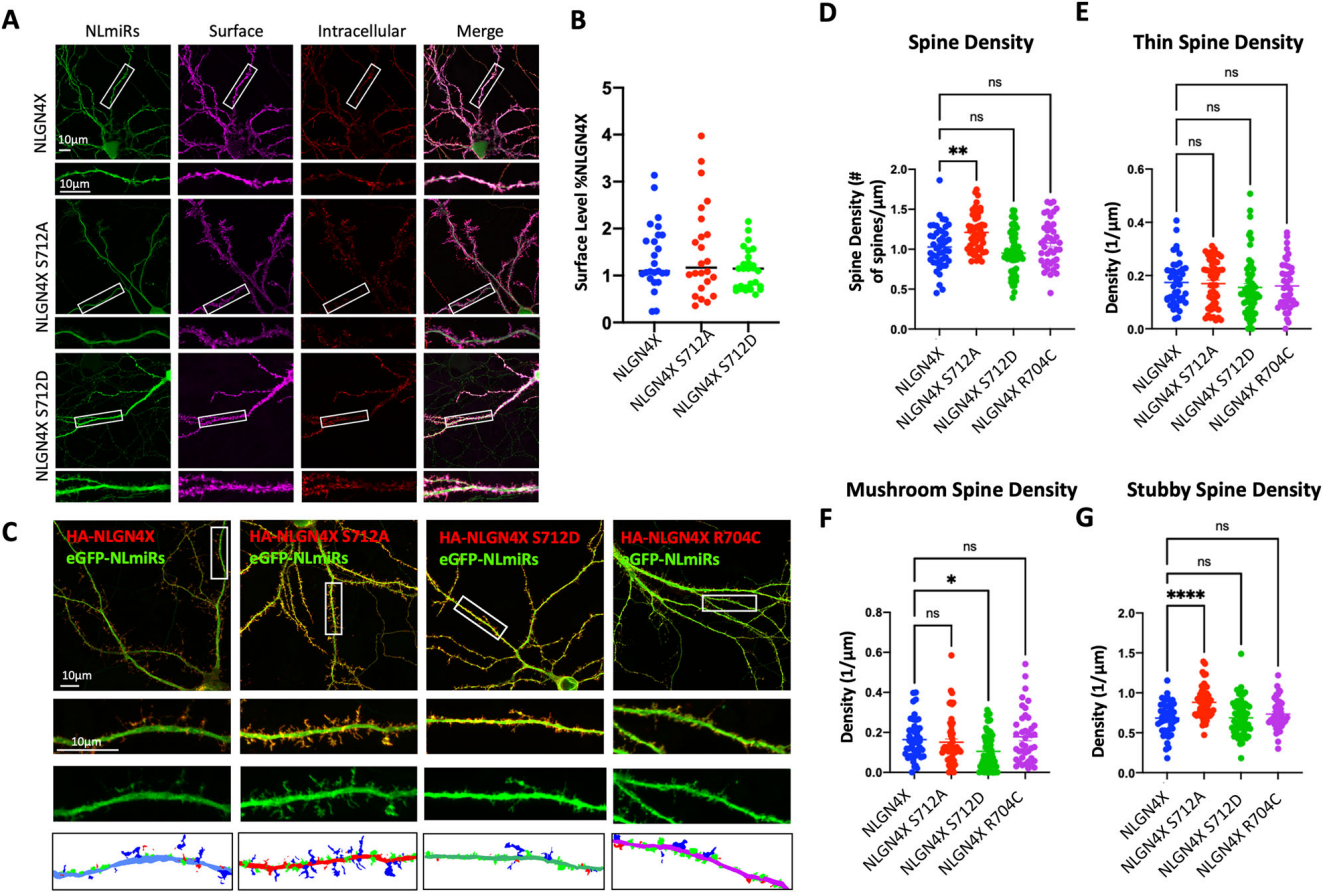
NLGN4X S712 phosphorylation affects spine density. ***A***, Coexpression of NLmiRs with wild-type, phosphonull (S712A), and phosphomimetic (S712D) mutants in cultured hippocampal neurons. Surface and intracellular NLGN4X was labeled with HA antibody, which recognized a tag inserted downstream of the signal peptide. Scale bar, 10 μm. ***B***, Mean ± SEM normalized to NLGN4X (*n* = 25), NLGN4X S712A (*p* > 0.05, *n* = 24), NLGN4X S712D (*p* > 0.05; *n* = 27). ***C***, Coexpression of NLmiRs with NLGN4X WT, NLGN4X S712A, NLGN4X S712D, and NLGN4X R704C transfected into cultured hippocampal neurons. Staining was done for total HA and GFP. Reconstructed dendritic spines in the bottom panels are color coded according to the spine type (red, thin; blue, mushroom; green, stubby). ***D*–*G***, Mean ± SEM of spine density (***D***), thin spine density (***E***), mushroom spine density (***F***), and stubby spine density (***G***) for NLGN4X (*n* = 44), NLGN4X S712A (*n* = 50), NLGN4X S712D (*n* = 57), and NLGN4X R704C (*n* = 44).

To characterize the effect of NLGN4X S712 phosphorylation, we compared the surface expression of NLGN4X (WT, S712A, or S712D) expressed in cultured rat hippocampal neurons and visualized with immunofluorescence confocal microscopy (Fig. 4*A*). To avoid the effects of endogenous NLGNs dimerizing with transfected NLGN4X, we cotransfected our cultured neurons with an exogenous chained microRNA against NLGNs 1, 2, and 3 (NLmiRs) as previously described (Shipman et al., 2011). Upon comparing the ratios of surface-to-intracellular NLGN4X WT with its phosphomimetic and phosphonull mutants, we did not observe any change in surface expression (Fig. 4*B*). Thus, we conclude that NLGN4X S712 phosphorylation does not directly affect NLGN4X surface trafficking.

We examined spine morphology to determine if NLGN4X S712A/D elicited synaptic changes despite unchanged surface expression. To quantity spine morphology, we optimized our protocol to use automatic spine detection in the neuron image analysis software, Neurolucida. Upon comparing spine densities of the various classifications of dendritic spines (mushroom, thin, stubby, or filipodia), we observed the phosphomimetic mutation of NLGN4X S712 (S712D) resulted in fewer mature mushroom spines as compared with NLGN4X WT and the phosphonull mutant (S712A; Fig. 4*F*). Interestingly we also observed a spinogenic effect in the phosphonull mutant, with NLGN4X S712A expressing markedly more total spines than the other conditions. This is in large part due to the significant increase in stubby spines found in the unphosphorylated condition (Fig. 4*D*,*G*). In summary, NLGN4X S712D decreases mushroom spine density compared with NLGN4X WT, consistent with fewer mature synapses. In contrast, NLGN4X S712A increases spine number, primarily through an increase in stubby spine density.

**Figure 5.**
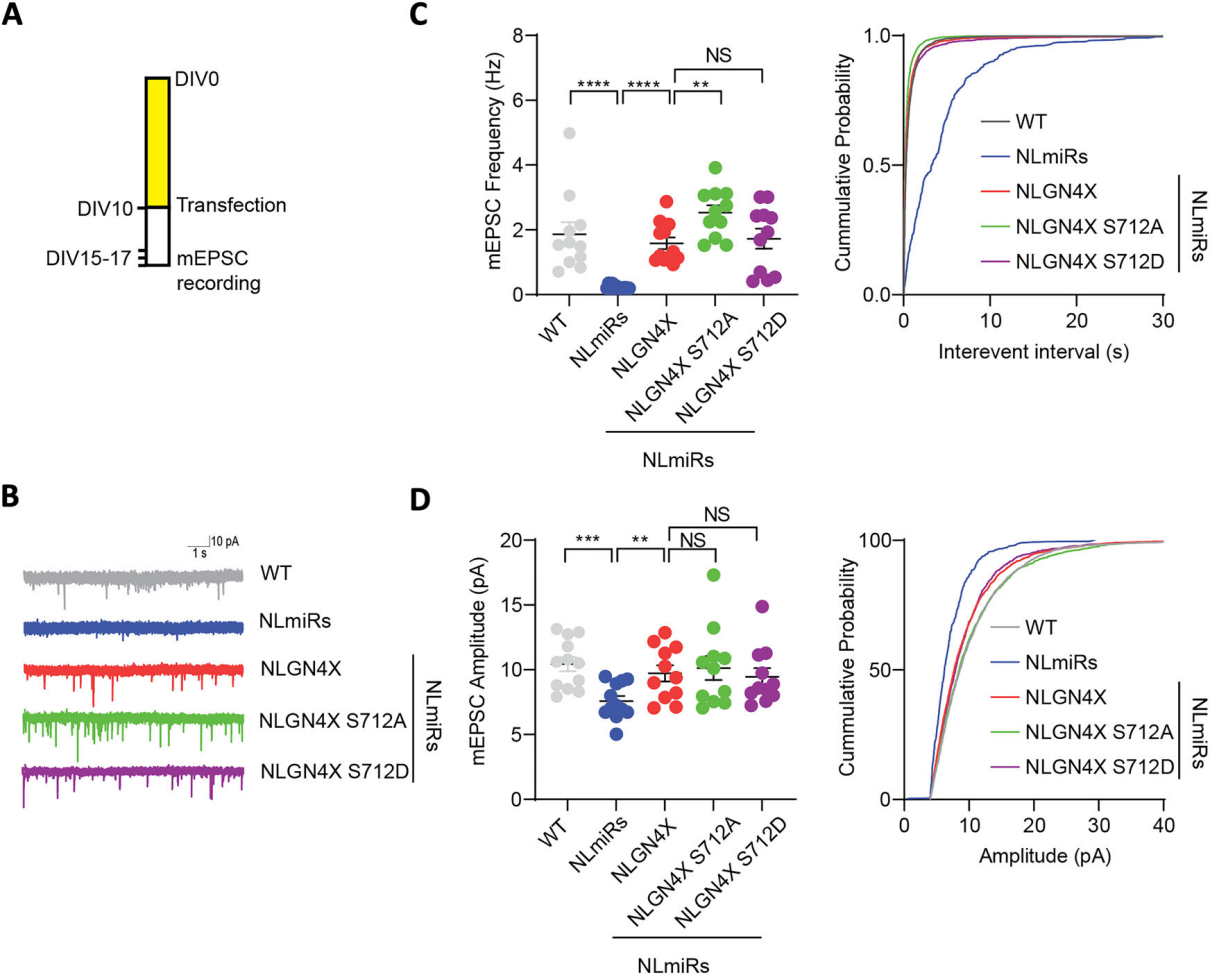
Phosphonull NLGN4X S712 enhances mEPSC frequency. ***A***, The experimental design for transfecting and recording from primary hippocampal rat neurons, as well as representative traces of mEPSCs recorded from those cells (***B***). ***C***, The mean of mEPSC frequency and cumulative probability plots of mEPSC interevent intervals and the mean of mEPSC frequency. *p* values were calculated using the Kolmogorov–Smirnov test with Dallal–Wilkinson–Lillie in WT (*n* = 12), NLmiRs (*n* = 14), NLGN4X WT (*n* = 11), NLGN4X S712A (*n* = 11), and NLGN4X S712D (*n* = 11) conditions. ***D***, The mean of mEPSC amplitude and cumulative probability plots of mEPSC amplitude and the mean of mEPSC amplitude. *p* values were calculated using the Kolmogorov–Smirnov test with Dallal–Wilkinson–Lillie in WT (*n* = 12), NLmiRs (*n* = 14), NLGN4X WT (*n* = 11), NLGN4X S712A (*n* = 11), and NLGN4X S712D (*n* = 11) conditions.

To establish the electrophysiological effects of NLGN4X phosphorylation, we recorded mEPSCs from hippocampal cultured neurons (Fig. 5*A*,*B*). These were transfected in the same manner as our immunocytochemistry experiments, cotransfecting NLGN4X, or its mutants, with NLmiRs to eliminate endogenous NLGNs, as well as a NLmiRs-only condition and a wild-type condition that was treated with Lipofectamine and no exogenous plasmids. As expected, transfection of only NLmiRs reduced both mEPSC frequencies and amplitude in primary culture hippocampal neurons. Consistent with our synaptic spine morphology data, the phosphonull NLGN4X S712A mutant increased mEPSC frequency (Fig. 5*C*). Both WT and phosphomimetic mutant showed no difference in mEPSC frequency or amplitude (Fig. 5*C*,*D*). Our electrophysiological studies, taken in conjunction with our spine analysis, indicate that phosphorylation of NLGN4X at S712 has a downregulatory effect on spinogenesis and spine maturation.

## Discussion

In this report, we identify a novel phosphorylation site on both NLGN4X and NLGN4Y at S712. NLGN4X can be robustly phosphorylated by both PKA and Cdk5, whereas NLGN4Y is robustly phosphorylated by Cdk5 but not PKA or PKC. We identify a single amino acid difference between NLGN4X (R710) and NLGN4Y (H710), which is important for the specificity of PKA phosphorylation of NLGN4X. We previously reported that NLGN4X phosphorylation via PKC in the ICD was disrupted by a familial case of ASD (Yan et al., 2005; Bemben et al., 2015a). Considering the proximity of this ASD-associated mutation (R704C) and the previously reported NLGN4X T707 PKC site, we also tested if NLGN4X S712 phosphorylation was disrupted by this ASD-associated mutation. We found PKA phosphorylation was not significantly impacted, despite the in vitro kinase assay data trending toward a reduction in phosphorylation, indicating a less dramatic but possibly biologically relevant reduction in PKA substrate affinity. Recent additions of unimpacted human variants in gnomAD call into question whether this disruption is or is not pathogenic. Upon using automatic dendritic spine classification, we show that the phosphomimetic version of NLGN4X S712 decreases spine maturation. In this paper, we reveal a novel mechanism for dynamically downregulating spine maturation via NLGN4X S712 phosphorylation, which is a target for both PKA and Cdk5.

We observed an increase in immature stubby spines with NLGN4X S712A expression, which in turn increases the overall average in total spine density. We also observed a decrease in mature mushroom spines upon expression of our phosphomimetic mutant, NLGN4X S712D. The NLGN4X R704C mutation does not show the same phenotype as the NLGN4X S712A mutation, despite also disrupting NLGN4X S712 phosphorylation. This could be potentially due to NLGN4X's adjacent phosphorylation site at T707, which has been shown to increase overall spine density and EPSCs and which are disrupted by the R704C mutation (Bemben et al., 2015a). Thus, NLGN4X R704C might disrupt the spinogenic effects of NLGN4X T707 phosphorylation while possibly simultaneously promoting the spinogenic effects of NLGN4X S712 phosphorylation, hence neutralizing any observable increase in spines. We should note that our study relies on overexpression of NLGN4X in various in vitro systems, so fully understanding the physiological implications of the regulation of this phosphorylation site in vivo requires further study. Unfortunately, because NLGN4X is a human-specific gene, it is difficult to study using in vivo models.

Advances in genetic sequencing of patient populations have helped identify candidate genes with pathogenic mutations implicated in ASD, these genes often fall into functional pathways critical for synaptic function (Jamain et al., 2003; Laumonnier et al., 2004; Yan et al., 2005; Singh and Eroglu, 2013; Kenny et al., 2014; Morris-Rosendahl and Crocq, 2020; Vieira et al., 2021). ASD, defined in The American Psychiatric Association's Diagnostic and Statistical Manual, Fifth Edition (DSM-5) by deficits in social interactions accompanied by restricted and repetitive behaviors, manifests with a broad range of phenotypes with often complex genetic etiology accompanied by environmental risk factors which predilects individuals toward ASD symptoms (fifth edition; DSM-5; American Psychiatric Association, 2013). A family study published in 2004 linked a frameshift mutation in NLGN4X to ASD in 13 patients across four generations (Laumonnier et al., 2004). In this study, only male family members were affected with severe intellectual disability (ID) and ASD, with female carriers having no discernable phenotype. The Simons Foundation Autism Research Initiative's curated gene database scores *NLGN4X* as a Category 1 high-confidence ASD–implicated gene. To date, many of the pathogenic variants on *NLGN4X* identified are frameshift mutations, nonsense mutations, and missense mutations in the extracellular domain, with only one studied missense mutation of an arginine substituted for cysteine (R704C) located in the ICD of NLGN4X (Yan et al., 2005; Bemben et al., 2015a; Chanda et al., 2016; Marro et al., 2019; Nguyen et al., 2020a). It is important to note this variant, from a male patient, had an unimpacted sister with the same mutation, and another sister who presented with ID, thus bringing the pathogenicity of this mutation into question. Additionally, this mutation does appear in gnomAD, an aggregated database of variants from unimpacted individuals, although most of these variants appear to be female and thus could potentially be carriers. In total, there are 21 missense variants listed in ClinVar found within the ICD of NLGN4X, with varying degrees of annotation and relevance to ASD, as opposed to 85 missense variants found within the ICD appearing in gnomAD (additionally, there are 360 vs 101 missense variants found within the ECD of NLGN4X in gnomAD and ClinVar, respectively, and 13 vs 4 in the transmembrane domain).

Recent studies indicate that the NLGN4X R704C mutation is a gain-of-function mutation, enhancing AMPA receptor EPSCs and resulting in greater affinity of NLGN4X to the AMPA receptor subunit GluA1 (Marro et al., 2019). We propose a model that the NLGN4X R704C mutation, which potentially disrupts NLGN4X S712 phosphorylation thus increasing GluA1 affinity, increases the number of AMPA receptors at the PSD. Conversely, phosphorylation of NLGN4X S712 could potentially decrease NLGN4X-facilitated AMPA receptor retention at the PSD, leading to a decrease in excitatory current. This model aligns with our observations of spine morphology, with phosphomimetic NLGN4X having an increase in stubby and overall spine density. The exact mechanisms whereby NLGN4X S712 regulates AMPARs have yet to be elucidated, and further studies are needed. Thus far, there is no literature showing a direct binding of NLGNs and AMPA receptor subunits.

Also of note are studies revealing that the intracellular portion of NLGN plays a role in actin remodeling as a way of modulating synaptic plasticity (Liu et al., 2016; Paskus et al., 2019). It was shown that NLGN1's interaction with the RhoGEF Kalirin-7 is an important regulator for spine dynamics, with in vitro results demonstrating that expression of both Kalirin-7 and NLGN1 are required for NLGN1's spinogenic phenotype typically observed in primary cultured neurons (Paskus et al., 2019). With the decrease of mushroom spines in the NLGN4X S712D phosphomimetic condition, it is possible that PKA and/or Cdk5 phosphorylation of NLGN4X regulates the interaction with a downstream RhoGEF or RhoGAP, promoting actin cytoskeletal remodeling of the synapse. While thin and mushroom spines are structurally similar, mushroom spines have a larger head with a thin neck and are more persistent (Gipson and Olive, 2017; Pchitskaya and Bezprozvanny, 2020). Thus, mushroom spines are thought to be sites of long-term storage, implicating NLGN4X S712 phosphorylation as a negative modulator for long-term memory. NLGN4X could also serve as a regulator of homeostasis, acting to dampen the strengthening of synapses in response to large influxes of Ca^2+^, or play a role in sleep, strengthening synapses when PKA levels are diminished (Hellman et al., 2010).

Specific mechanisms that direct downregulation of spine maturation upon phosphorylation of NLGN4X remain an important line of inquiry. Typically, activity-induced kinases have upregulatory effects on synaptic transmission. Our observed phenotype has an opposite effect, opening alternative avenues for the physiological importance of this phosphorylation site, and more broadly expands our understanding NLGN4X's role at the synapse.

## Synthesis

Reviewing Editor: Mei Zhen, University of Toronto Decisions are customarily a result of the Reviewing Editor and the peer reviewers coming together and discussing their recommendations until a consensus is reached. When revisions are invited, a fact-based synthesis statement explaining their decision and outlining what is needed to prepare a revision will be listed below. The following reviewer(s) agreed to reveal their identity: Xavier Altafaj, Dhrubajyoti Chowdhury.

SYNTHESIS

Both reviewers found the work of relevance. However, particularly reviewer # 2 raised questions requiring additional experiments such as evidence for phophorylation of endogenous NLGN4X or controls for the effects of PKA activation. They also noted formal mistakes, lack of detail in experimetns or replicate numbers that need to be corrected.

REVIEWER #1:

The authors of the manuscript entitled "Activity-dependent phosphorylation of NLGN4X regulates spinogenesis and synaptic function" are addressing an important question in the field of neuronal development and circuit formation, namely the role of cell-adhesion molecules (i.e. neuroligins) and their modulation by post-translational modifications (i.e. phosphorylation). The work is focused on the delineation of NLGN4X, which is relatively non-conserved between human and mouse, and thus represents a technical challenge.

In the abstract section, the sentence referring to ASD-associated mutations should be removed, since the association between phosphorylation and integrated / developmental dimension are not defined yet. Indeed, throughout the manuscript the authors should focus on the data-based conclusions provided by the elegant biochemical, cellular and electrophysiological data provided, while limiting the speculative hypothesis of an association between NLGN4X phosphorylation and ASD, which should be limited to the discussion section.

This is an interesting manuscript, providing new and relevant biochemical insights on the phosphorylation events that regulate neuroligins activity, with a particular focus on NLGN4X. The manuscript requires additional experiments (biochemistry and cell biology), together with a necessary proof-reading (including figure legends) to avoid overstatement (ASD link is probably too speculative and should be restrained to the discussion section).

Results:

Figure 1A should be described in the results section, to show previous knowledge on phosphosites and present the aim of the in vitro kinase experiments.

Figure 1B, and western blot images: please indicate with an arrow the expected molecular weihgt of the fusion proteins (non-degradated). The autoradiography of CamKII-mediated phosporylation assay shows a signal on NLGN4X (not for 4Y), and thus the sentence "We

observed that PKC and PKA robustly phosphorylated NLGN4X, but not 4Y, whereas CaMKIIα phosphorylated neither" is not correct.

Please provide the sequence and hits (peptides number) of pS712-containing phosphopeptides identified using LC-MS/MS. Is this serine 712 residue the only amino acid identified using mass spec? This can be interesting, to know the extent of the phosphorylation profile / substrates of NLGN4X-ICD.

Figure 2: the authors claim that "we showed that PKC-mediated phosphorylation of NLGN4X T707 and PKA-mediated phosphorylated NLGN4X S712 are highly specific (Figure 2A-D). The experiments in figure 2 show the kinase activity of specific kinases on different phospho-deficient mutants. To fully support the sentence, the authors should do the experiments using the same mutants panel (e.g. T707A + PKA; T712A + PKC).

The functional studies in primary neuronal cultures are elegantly designed, interesting, and support the conclusions of both morphological (spines) and electrophysiological changes resulting from NLGN4X-S712 phosphorylation modulation. Nevertheless, considering the primary role of NLGN4X (cell-adhesion molecule, interacting with NRX), the manuscript would benefit from investigating the formation of mature synapses. This is important to assess the in vitro functional relevance of this phosphosite, to know whether synapses (e.g. vGlut-PSD95 positive puncta double immunofluorescence technique might be considered, or another approach).

Formal aspects (relevant): Figure legends are neither self-explaining, nor precise (e.g. Fig. 3). Please proofread the figure legends to allow reader's understanding of the results. Make the figure legends consistent (statistics description)

Minor points:

Introduction:

- Gene names should be written in italics

- Besides ASD core clinical symptoms include the mentioned impairments of communication and social interaction, according to the Diagnostic and Statistical Manual of Mental Disorders (DSM-5), people with ASD often have restricted interests and repetitive behaviours.

- The sentence "Advances in genetic sequencing of patient populations have helped identify candidate genes with many harboring mutations pathogenic for ASD" should be rephrased, with more precision and grounded on bibliographic references and SFARI gene categories description. More importantly, a statement presenting the genetic inheritance complex genetic origin, as well as the role of environmental factors associated with ASD should be mentioned. This is a key point while talking about ASD.

Please provide the number of neutral vs. disease-associated variants (for the different domains), while describing " To date, most of the pathogenic variants on NLGN4X identified are frameshift mutations, nonsense mutations, and missense mutations in the extracellular domain, with only one missense mutation of an arginine substituted for cysteine (R704C) located in the intracellular domain (ICD) of NLGN4X". This information will put in context the relevance of investigating missense mutations of the ICD. An updated version of the reported disease-associated variants might be retrieved from ClinVar, while neutral variants might be found in gnomAD. In particular, the authors should revise the statement about R704C variant: since it is not described in ClinVar and appears in gnomAD, it is likely a non-pathogenic genetic variant.

Figure3A: the merged image for S712A should be centered. Please indicate the pesudocolor channels for PSD95 and HA-NLGN4X constructs

Supplemental Figure 1, panel B: Do the authors performed the Ig negative control (ideally using pre-immunized serum) in the same experiment? Please note that, probably during the process of PDF formating, the anti-NLGN4X western blot was shifted and needs to be corrected.

Methods: Primary neuronal cultures are subject to variability which is also influenced by the complex genetic manipulations (cotransfection). Please indicate the number of experiments, cells / experiment / dendrite / cell and spines / cell considered for the spines density experiments.

REVIEWER #2:

The current study identified a phosphorylation site within the intracellular domain of neuroligin4X (NLGN4X), a human-specific isoform of NLGN family of adhesion molecules, and examined the effect of phospho-mutants on dendritic spines and synaptic transmission. Through vitro assays using purified recombinant proteins and heterologous expression systems, S712 was identified as a target of PKA and Cdk5. NLGN4X harbors autism-linked mutations (e.g. R704C) and therefore, studying its post-translational modifications (PTMs) and their effects on synapse structure/function is significant. However, although the present study attempts to reveal such significant molecular details, its conclusions are not adequately supported by the data in this manuscript. Importantly, descriptions of the results, methods, figure legends, quantification need serious revision; this manuscript is sketchy on the details, sometimes inconsistent, making it challenging to evaluate the findings. Below are the details of the major concerns that need to be addressed:

1. Since NLGN4X-S712 phosphorylation is central to this study and was claimed to be driving synaptic changes, it is crucial to establish that endogenous NLGN4X is phosphorylated at S712. The pS712 antibody was used to detect this phosphorylation in in vitro kinase assays for the recombinant NLGN4X and validated using S712A mutation in this context. This antibody was used for immunoprecipitation from iPSC-derived neurons and immunoprecipitates blotted for NLGN4X to detect endogenous S712 phosphorylation (Suppl. Fig. 1A,B). However, this result is not convincing as the same antibody can bind phospho-NLGN4Y (Fig. 2C,E), which is expected given the epitope is conserved between the two isoforms (Fig. 1C). Moreover, given the residual signal in S712A-NLGN4X (Fig. 1D), the pS712 antibody might immunoprecipitate the unphosphorylated protein, which will likely account for majority of the NLGN4X in neurons under the non-stimulated conditions. In order to establish bonafide S712 phosphorylation of endogenous NLGN4X, reverse IP from these neurons should be done with NLGN4X antibody and IP complexes probed with pS712 antibody and enhanced phosphorylation demonstrated by activity induction or PKA activation. Notably, no activity manipulation in neurons was performed in this study and, therefore, use of the term "activity-dependent phosphorylation" in the title seems unjustified.

2. The effect of R704C mutation on NLGN4X-S712 phosphorylation (Suppl. Fig 1C,D) needs clarification. PKA activation by forskolin (FSK) was used to study overexpressed NLGN4X phosphorylation. Since there was no difference in phosphorylation upon FSK treatment, how do the authors know whether PKA is activated in their experiments? Does no difference between -/+ FSK mean PKA is not the kinase for NLGN4X in situ? A positive control for FSK action is necessary to evaluate the validity of this assay. Importantly, pS712 signal for R704C mutant is reduced compared to WT even without FSK. As the total IP levels for R704C are also reduced compared to WT (HA blot), the lower pS712 signal can be simply due to less stability or lower expression of this mutant, and independent of phosphorylation per se. No difference between -/+ FSK also raises doubt about the phospho-specificity of the pS712 antibody used in this study, which might detect unphosphorylated NLGN4X as long as the epitope region used to raise the antibody remains intact. Loss of pS712 signal in S712A mutant might be due to disruption of critical epitope recognition motif (Fig. 1C). Phosphatase treatment should be done to demonstrate the phosphorylation dependence of the antibody. Overall, the interesting conclusion of an autism-linked mutation regulating NLGN4X PTM is not supported by the current evidence. Moreover, this mutation does not seem to have any effect on spine morphology as shown by the authors (Fig. 3C-G).

3. For the spine morphology experiments (Fig. 3C-G), I assume PSD-95 immunostaining was used to label spines, though it is not clearly stated in the methods section nor in the figure legends (need to be revised thoroughly, even figure panels not labeled for proteins!). If so, it is not an appropriate marker to classify spine morphological subtypes because PSD-95 is largely absent from immature spines and is developmentally upregulated to be found predominantly in mature/mushroom spines. Therefore, there will be obvious bias in sampling if such labeling was used for morphological classification leading to inaccurate quantification. Standard practice is to use a cytosolic soluble fill to visualize dendritic protrusions, such as the GFP expressed with the NL-miRs used in these experiments. What was the effect of expression of the NLGN4X mutants on PSD-95 synaptic levels? This will be interesting to know to assess the effect on excitatory synapses which would complement the functional mEPSC data.

4. The statistical analyses for the cumulative probability plots comparing the different conditions are missing in the mEPSC data in Fig. 4. What does WT condition refer to in this assay? Figure legend says cortical neurons were used while hippocampal neurons stated in main text. Which is correct? What does n refer to? The figure legend is inconsistent with the data points on the graphs.

5. What statistical test was used in Fig. 3? What does n refer to? NLmiRs expression reported by GFP looks very different between the different conditions (Fig. 3A). Different levels of knockdown of the endogenous NLs will allow different degrees of heterodimerization with the ectopically expressed NLGN4X and will have direct influence on its surface trafficking (Fig. 3B), thereby confounding the effects of the point mutations studied. How did the authors correct for such discrepancies in endogenous NL levels? Also, the assay protocol and quantification need to be described in details.

6. The numbering of the NLGN4X and 4Y aligned sequences seems wrong (Fig. 1C).
